# New complete genome sequences of human rhinoviruses shed light on their phylogeny and genomic features

**DOI:** 10.1186/1471-2164-8-224

**Published:** 2007-07-10

**Authors:** Caroline Tapparel, Thomas Junier, Daniel Gerlach, Samuel Cordey, Sandra Van Belle, Luc Perrin, Evgeny M Zdobnov, Laurent Kaiser

**Affiliations:** 1 Central Laboratory of Virology, Division of Infectious Diseases, University of Geneva Hospitals, 24 Rue Micheli-du-Crest, 1211 Geneva 14, Switzerland; 2 Department of Genetic Medicine and Development, University of Geneva Medical School, 1 Rue Michel-Servet, 1211 Geneva 14, Switzerland; 3 Swiss Institute of Bioinformatics, 1 Rue Michel-Servet, 1211 Geneva 14, Switzerland; 4 Imperial College London, South Kensington Campus, SW7 2AZ London, UK

## Abstract

**Background:**

Human rhinoviruses (HRV), the most frequent cause of respiratory infections, include 99 different serotypes segregating into two species, A and B. Rhinoviruses share extensive genomic sequence similarity with enteroviruses and both are part of the picornavirus family. Nevertheless they differ significantly at the phenotypic level. The lack of HRV full-length genome sequences and the absence of analysis comparing picornaviruses at the whole genome level limit our knowledge of the genomic features supporting these differences.

**Results:**

Here we report complete genome sequences of 12 HRV-A and HRV-B serotypes, more than doubling the current number of available HRV sequences. The whole-genome maximum-likelihood phylogenetic analysis suggests that HRV-B and human enteroviruses (HEV) diverged from the last common ancestor after their separation from HRV-A. On the other hand, compared to HEV, HRV-B are more related to HRV-A in the capsid and 3B-C regions. We also identified the presence of a 2C *cis*-acting replication element (*cre*) in HRV-B that is not present in HRV-A, and that had been previously characterized only in HEV. In contrast to HEV viruses, HRV-A and HRV-B share also markedly lower GC content along the whole genome length.

**Conclusion:**

Our findings provide basis to speculate about both the biological similarities and the differences (e.g. tissue tropism, temperature adaptation or acid lability) of these three groups of viruses.

## Background

Human rhinovirus (HRV) is the most frequent cause of infection across all age groups of the population [1]. Replication is often restricted to the upper respiratory tract leading to self-limited illnesses such as the common cold. However, HRV infections can also exacerbate pre-existing airway disorders, invade the lower respiratory tract and lead to serious complications [2,3].

HRVs are single positive-stranded RNA viruses of approximately 7200 base pairs. They belong to the *Picornaviridae *family and are closely related to HEVs, another genus of the same family. The genome organization of *Picornaviridae *is conserved among the family with a long 5'-untranslated region (UTR), a single open reading frame (ORF) encoding a polyprotein, a short 3'UTR, and a poly(A) tail [4]. The 5'-terminal UMP of the viral RNA is covalently linked to the small viral protein VPg [5]. The 5'UTR contains two structural elements [6]. One is the 5'-cloverleaf structure involved in the plus-strand RNA synthesis and in the process of switching from translation to replication [7,8]. The other is the internal ribosomal entry site (IRES) which promotes translation of the polyprotein. The 3'-UTR is necessary for efficient RNA replication, but the exact mechanism is still not well understood [9,10]. In addition, species-specific internal *cis*-acting replication elements (*cre*) have been identified in HEV [11,12], HRV-A [13] and HRV-B [14,15].

HRV strains have been classified into 99 serotypes [16] based on the ability of a given serum to neutralize virus growth of a given strain in cell culture, although several serotypes share significant antigenic cross-reactivity [17]. According to nucleotide sequence relatedness of some serotypes [18-21] and to sequence comparison of all serotypes in the VP1 [16,22] and VP4-VP2 capsid protein-coding regions [23], the 99 serotypes segregate in two different groups: 74 belong to the HRV-A species and 25 to the HRV-B species. In addition to the division of HRVs into two species, they have also been classified into major and minor groups according to receptor usage. The major group ofHRVs (composed of 65 serotypes of species A and 25 serotypes of species B) binds ICAM1, whereas the minor group viruses (9 serotypes of species A) bind preferentially to LDL receptors [24-27]. The existence of multiple serotypes within each of these two lineages and different receptor usage support the hypothesis of significant differences at the protein level. Surprisingly, despite the fact that HRVs are the major cause of human respiratory infections, little is known about their genome variability at the full-length scale. To the best of our knowledge, part of the VP4-VP2 [23] and VP1-2A [16,22,28] regions have been sequenced for all serotypes and half of them for the 3D regions [19], but full-length sequences of only 8 serotypes are publicly available in the *Picornaviridae *database [29] (7 HRV-A and 1 HRV-B) [30-37]. While the present manuscript was in the process to be accepted, Kistler and coworkers published additional HRV-A and HRV-B full-length sequences increasing significantly the number of sequences available [38].

Among the *Picornaviridae*, HEVsare the closest relatives of HRVs and, as for HRVs, humans are the only known reservoir. Phylogenetic analyses of VP1-2A HRV and HEV sequences suggest thatHRVs and HEVs could be considered members of the same genus [28]. In addition, HRV-87 presents a high sequence similarity to HEVs and was recently reclassified as EV-68 [18,23,39,40]. Yet, the exact relation between HEV and HRV remains ambiguous without full-length genome comparison. At the phenotype level, however, HRV and HEV are clearly distinct: *in vitro*, cell tropism, pH tolerance and optimal growth temperature are significantly different; *in vivo*, the site of infection, organ tropism and the ability to disseminate are well-established characteristics that differentiate HEVs from HRVs. HRVs infections are restricted to the respiratory tract (temperature of 33°C), whereas most HEVs have the ability to replicate predominantly in the gastrointestinal tract (37°C). A large proportion can also disseminate, causing viremia and potentially invading the central nervous system [41]. Full-length genome comparison of these two genera helped us to identify genomic features and divergences at the amino acid level that might explain some biological differences.

We have sequenced 12 full-length genomes of different HRV serotypes and we present here the comparative analysis of 20 prototype HRV strains (13 HRV-A and 7 HRV-B) and 14 publicly available HEV strains that identifies the key elements differentiating these medically important viruses.

## Results

The 12 newly sequencedHRV genomes have been deposited in GenBank [GenBank accession EF173414–EF173425]. They vary in sequence length from 7124 nucleotides (HRV-12) to 7219 nucleotides (HRV-17) which is similar to the length range of previously sequenced genomes (from 7102 to 7208 nucleotides). The average size ofHRVs type A (7131 nt) is smaller than the average size of HRVs type B (7215 nt), whereas the average size of 14 HEVs analysed in this study is 7417.

### HRV-B is more closely related to HEV than to HRV-A

#### Phylogenetic analysis

**Figure 1:**
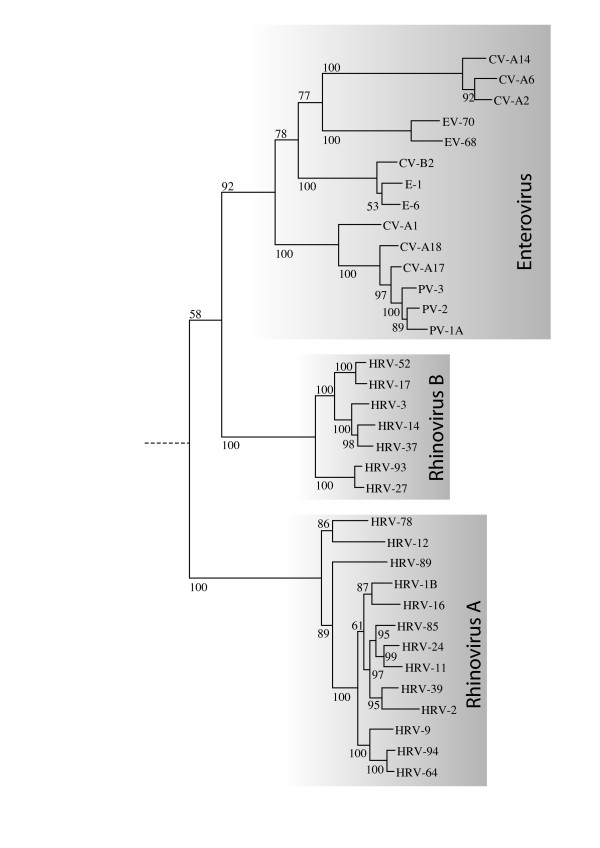
**Whole-polyprotein phylogenetic tree**. Whole-polyprotein, maximum likelihood phylogenetic tree shows a closer relation between HRV-B and HEV than between HRV-A and HRV-B. The figure indicates the percentage of bootstraps (out of 1000) that supports the corresponding clade. The sequence of simian picornavirus 1 (SV-2) was used as an outgroup. The branch lengths are measured in substitutions per site.

**Figure 2:**
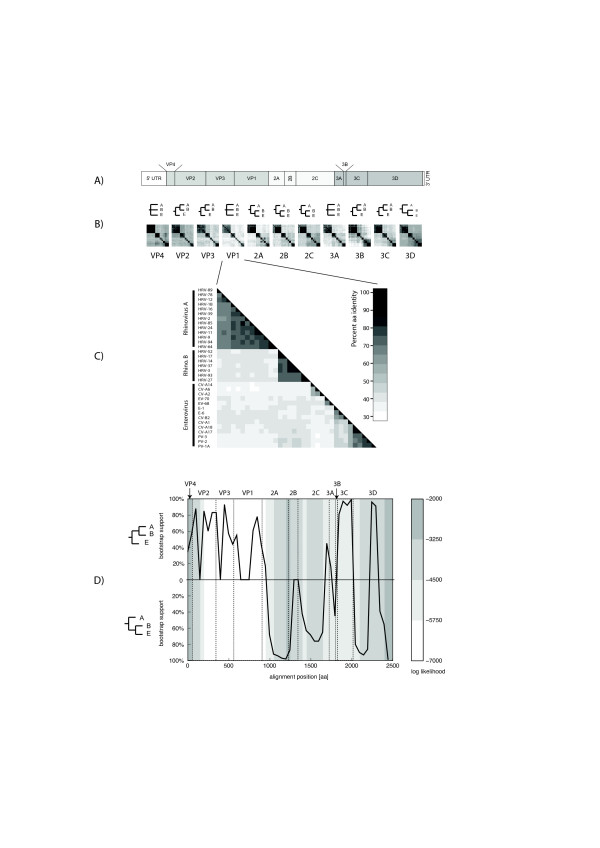
**Protein and amino-acid similarity comparison between HRV-A, HRV-B and HEV**. A) Schematic representation of HEV and HRV genome organization showing boundaries of encoded proteins. B) Protein similarity comparison. For each protein, the following is shown: – top row: a simplified tree representation of the relationships between HRV-A (A), HRV-B (B) and HEV (E), according to the corresponding ML tree (see additional file 1). Three cases are possible: HRV-B closer to HRV-A; HRV-B closer to HEV; and undecided (none of the above clearly more likely than the other). – bottom row: an all-versus-all sequence identity matrix (darker colorus indicate higher identity percentage). The similarity values are given in additional file 2. C) Close-up of the identity matrix for VP1. D) Bootscanning. The whole polyprotein alignment was divided into windows of 200 aa starting every 50 aa, and a 100-bootstrap ML tree was computed on each window. The black curve indicates the degree of support (as a percentage of bootstrap replicates) for either the "HRV-B closer to HRV-A" (upper half) or "HRV-B closer to HEV" (lower half) topology at each position along the whole genome (see Methods for details). The background colour reflects the log likelihood of the tree at each position which is a measure of overall confidence in the tree. Darker colour indicates higher confidence.

The phylogeny of HRV-A, HRV-B and HEV was reconstructed by using maximum-likelihood phylogenetic method for the full polyproteins (Figure 1), as well as for each individual protein, using Simian picornavirus (SV-2) as the outgroup. The whole-polyprotein phlyogenetic analysis suggests the hypothesis that HRV-B and HEV lineages radiated from a common ancestor after its separation from HRV-A (Figure 1), where the percentage of bootstrap support of each grouping reflects the statistical confidence. Yet, when the analysis is conducted at the level of each individual protein, the subsequent reconstructed tree topology does not always support the same conclusion (Figures 2B-D and additional file 1). In the region of VP2, VP3, 3B and 3C proteins, the analysis supports the alternative hypothesis that HRV-B and HRV-A radiated from a common ancestor after separation from HEV. In the region of VP4, VP1 and 3A proteins, the analysis cannot discriminate the phylogenetic relationships between these three virus groups. The bootscanning experiment (see Methods) presented in Figure 2D is consistent with all the above-mentioned findings and also supports the hypothesis that in some parts of the capsid HRV-A and HRV-B share the last ancestor after their separation from HEV.

#### Protein product similarity

For each individual protein cleavage product, we also quantified pair-wise sequence identities among the HRV-A, HRV-B, and HEV genomes under consideration. Figure 2(A-B-C) shows the arrangement of these proteins along the picornavirus genomes and the corresponding protein identity matrices. Over all these three picornavirus (HRV-A, B and HEV), VP1 is the least conserved protein (48.5 % amino-acid identity) and VP4 the most conserved (66,6%). Globally, these comparisons are consistent with the phylogenetic analysis and confirm that the 2A, 2B, 2C, and 3D percentage of sequence similarities are higher between HRV-B and HEV than between HRV-A and B (see additional files 1 and 2). In contrast, at the level of VP1, VP2, VP3, 3B and 3C sequences, HRV-A and HRV-B have a higher percentage of homologies compared to HEV (see additional files 1 and 2).

Between the HRV-A and HRV-B groups, VP2 shows the highest overall conservation (> 60%) and 2A protein exhibits the lowest (< 40%). Within each of the species (HRV-A or HRV-B), the VP1 protein appears as the least conserved (< 80% of averaged amino-acid identity) and VP4 as the most conserved (> 96%) (see additional file 2). Protein 3B also shows poor conservation among HRV-B serotypes, but this may be an artefact due to its small size.

### RNA structural elements

#### 5'- and 3'- UTRs

Analysis of the 5'UTR of HRV-A, HRV-B and HEV shows that the well known 5' cloverleaf *cre *element as well as the IRES structure are highly conserved throughout the three groups (see additional file 3). The cloverleaf structure was originally discovered in polioviruses [42]. This secondary structure is deposited in the Rfam database (a collection of multiple sequence alignments covering many common non-coding RNA families and conserved RNA secondary structures [43]) under the accession number [RF00386]. The corresponding consensus structure of HRV-A, HRV-B and HEV, which was recovered without prior knowledge of it by comparative sequence analysis (see additional file 3A) matches well this Rfam consensus structure. In the same line, the IRES structure for HRV-A, HRV-B and HEVs is also very similar to the Rfam *Picornaviridae *consensus structure [RF00229] [44], except for the presence of two additional small helices (see additional file 3B).

The picornavirus 3'-UTR encodes astem-loop structure that may play a role in replication efficiency (through interaction with the 5'UTR) as well as in polyadenylation of genomic RNA [9,45,46]. In contrast to the 5'UTR structures, the 3'UTRs structures (see additional file 4)are not universally conserved in sequence and position among the three groups studied. The length of the 3' UTR between the groups varies between 47 nt (HRV-A), 50 nt (HRV-B) and 83 nt (HEV). Furthermore, both HRV-A and HRV-B contain a stable stem-loop structure of 35 nt at the 3' end of the 3'UTR. HEV also contains a 42 nt stem-loop which is located closer to the middle of the 3'UTR. Nevertheless, there is a large amount of sequence variability within this whole group which leads to a less stable consensus stem-loop for all the analyzed HEV sequences.

In addition, there is a conserved stem-loop structure in HRV-A located close to the 3'UTR, yet the corresponding region in HRV-B and HEV suggest different structures, and the overall high sequence conservation in the region could give a misleading signal of structural conservation (see additional file 5).

#### Internal cis-acting elements

Besides the 5' and 3'UTR, disparate internal *cre *elements have been previously described among various rhinoviral serotypes of both HRV-A and HRV-B [15], and have been identified by our comparative analysis.

##### HRV-A *cre*

The HRV-2 2A internal *cre *motif [Rfam RF00220] [13] is conserved among all HRV-A genomes analysed in this study, but has not been identified in any HRV-B or HEV viruses (see additional file 6A). The same region of HRV-B also folds into a conserved secondary structure that seems specific to this group (data not shown).

##### HRV-B *cre*

Similarly, the internal *cre *motif reported for the HRV-14 VP1, a member of HRV-B, is present in all 7 HRV-B serotypes and is notably absent in all HRV-A and HEV analyzed (see additional file 6B).

**Figure 3:**
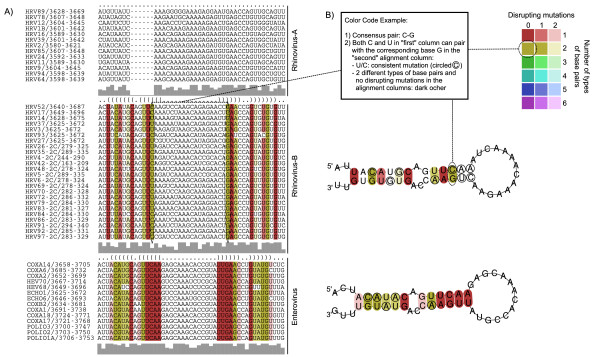
**Alignments and conserved secondary structures for cis-acting 2C replication elements conserved within HRV-B and HEV**. A) Multiple sequence alignment across all considered genomes that shows consensus secondary RNA structure (in dot bracket format, see first row); sequences are colour-coded according to RNA structure conservation; the sequence conservation profile for each group is shown in grey bars beneath the alignments. B) Secondary structure of the conserved *cre *2C, colour-coded according to the different types of base pairs in the corresponding alignment columns. The more different the types of base pairs existing for two pairing alignment columns, the more evolutionary conservation of the structure (cp. compensatory and consistent mutations).

Furthermore, the availability of new HRV-B sequences allowed us to identify another conserved *cre *motif within the HRV-B 2C coding sequence (Figure 3) that has the typical R_1_NNNAAR_2_NNNNNNR_3 _*cre *motif [47-51] in all HRV-B serotypes analysed (the 7 full genomes plus 17 partial sequences), with the exception of HRV-27 that has a U instead of an R at position R_1_. More importantly, the newly identified HRV-B 2C *cre *corresponds to the HEV 2C *cre*, previously identified in several HEVs [11,12].

### GC content

**Figure 4:**
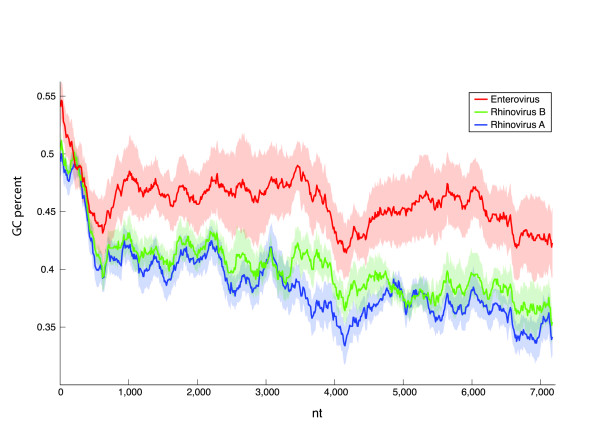
**Local GC composition of HRV-A, HRV-B, and HEV**. Average GC percentage computed over a sliding window of 600 nt and a step of 10 nt along whole-genome multiple alignments of HRV-A, HRV-B, and HEV, respectively (thick lines). The shaded areas represent one standard deviation above and below the average.

The GC composition is an important genomic factor that can be evolutionary optimized for adaptation to multiple environmental constraints (such as ideal growth temperature). The GC content varies substantially between the groups of HEV, HRV-A and HRV-B (Figure 4), where HRV-B exhibits lowest values, HEV exhibits the highest values, and HRV-B is intermediate. This holds not only globally, but also locally, for each of the sliding windows along the whole genomes. These trends are statistically significant as the two-sided Kolmogorov-Smirnov test rejects the hypothesis that GC contents of HRV-A, HRV-B and HEV can be drawn from the same underlying distribution: HRV-A vs. HRV-B p-value < 10^-15^; HRV-A vs. HEV p-value < 10^-15^; HRV-B vs. HEV p-value < 10^-15^.

## Discussion

HRVs were first classified into two groups based on a differential sensitivity to a variety of antiviral compounds targeting VP1 [52]. The members of the HRV-A group were susceptible to most of these antiviral compounds, whereas the HRV-B were not. This classification was then confirmed by nucleotide sequence relatedness in the VP1 [16,22] and VP4-VP2 capsid protein-coding regions of all serotypes [23]. Analysis of other regions like the 3C protease has been restricted to a limited number of serotypes [18,20,21]. Whole genome comparisons have not been conducted since only one full-length HRV-B genome (HRV-14) as well as a limited number of HRV-A genomes were available. Complete sequencing and analysis of additional HRV-B and HRV-A genomes allowed us to describe their phylogeny and the similarity of individual proteins between the two HRV groups and HEV. For example, proteins such as 2A show a particularly pronounced difference in inter- versus intra-group conservation. Conversely, surface proteins such as VP2 (capsid) are better conserved across all groups.

It appears that HRV-B share a common ancestor with HEV as shown by the whole-genome phylogenetic analysis, which suggests that Rhinovirus is not monophyletic. This observation is reinforced by the identification of a new HRV-B 2C *cre *that is lacking in all HRV-A genomes studied. This *cre *consists of a hairpin structure with a conserved R_1_NNNAAR_2_NNNNNNR_3 _motif in the loop [47-51] and was previously only known in HEV 2C gene. The first two As in this motif serve as the template for the VPg uridylylation reaction by the viral polymerase. Uridylylated VPg then serves as a primer for RNA synthesis [5]. Although the question is still open to debate, it has been suggested that the polyA tail may serve as template for VPg uridylylation and synthesis of the minus strand RNA, whereas internal *cre *are necessary for plus strand synthesis [53,54]. HRV-14 VP1 *cre*, as well as HRV-2 2A *cre*, were shown to be functionally equivalent to poliovirus RNA 2C *cre *in *in vitro *uridylylation assays with poliovirus VPg and polymerase [11-13,49]. Further studies are underway to define whether the HRV-B 2C *cre *identified in this study plays a role in VPg uridylylation or can be considered as an evolutionary leftover of HEV 2C *cre*. Concerning the replacement of an R position by a U in HRV-27 *cre*, it should be noted that the effect of a similar substitution on replication efficiency could not be studied for HRV-14 2C *cre *since it results in the introduction of a stop codon [47-51]. However, for Coxsackiesvirus B3 2C *cre*, a substitution at the R3 position by a U was shown permissive for replication [53,54].

Besides this putative new *cre *element in the 2C region of HRV-B, we could also identify the already known elements (cloverleaf structure and IRES in 5' as well as the stem-loop element in 3') in the 5' and 3' UTRs from all studied genomes. The cloverleaf structure and the IRES are highly conserved. Interestingly, we identified many compensatory mutations in the sequences of these structures, which points out that the selective pressure is "working" on the structural level. The functionality of these elements is therefore more determined by their structure than by their primary sequence.

The observation that HRV-B and HEVs are more closely related to each other than either is to HRV-A seems paradoxical, given that HRVs differentiate themselves from most HEVs at the phenotypic level. This could be explained by recombination events that would have taken place soon after the divergence of HEV and HRV-A and during which regions were exchanged between HRV-A and the HEV ancestor of HRV-B. The protein identity plots and the bootscanning suggest that the recombining region may have included the capsid region. This is consistent with the fact that recombination breakpoints have been found to be largely restricted to nonstructural regions of the genome, mostly in the 2A-2C parts, and between the 5' UTR and the capsid-encoding region [55-57]. Recombination has been extensively documented as a driving force for the evolution of some *Picornaviridae *[58], although only hypothesized forHRVs [19]. Interspecies *in vivo *recombination was also suspected for HEVs [59]. Our hypothesis is that early recombination events may have occurred between HRV and HEV. Although these two virus species have often different tropism *in vivo*, both can easily infect the respiratory tract, an event that could provide opportunities for recombination.

Since VP1 is responsible for recognizing the receptor on the host cell surface, the hypothesis of capsid sequence transfer from HRV-A to HEV to yield HRV-B could explain a tissue tropism and a disease pattern similar to that of HRV-A rather than HEV. In addition, the similar GC content observed between HRV-B and HRV-A may account for some of these phenotypic differences. The relatively lower GC content of HRV-B may reflect an adaptation to the environment of the upper respiratory tract, whereas the higher GC content of HEVs might reflect convergent adaptation to the gastrointestinal tract of the central nervous system environment (such as higher temperature, acidity, etc.).

## Conclusion

The analysis of new HRV full-length genomes statistically supports (> 90% bootsrap confidence) the current classification of HRV into two distinct species. HRV-B seems to be phylogenetycally more closely related to HEV, another important member of the *Picornaviridae *family, than it is to HRV-A. However, our observations suggest that this species classification accurately reflects the capsid type, but not all parts of the genome. We have shown that HRV-A and HRV-B differ significantly at the protein level and in the composition and structure of their *cis*-acting sequences. One of the evolutionary scenarios that would explain the differential grouping of HRV-B with HEVs or with HRV-A along the genome is that of an ancient recombination between HRV-A and HEV lineages, given that the HRV-B closer relation with HEVs is overall more statistically sound. However, without additional data, this remains only a hypothesis. The genomic features highlighted in our study help to contribute to our understanding of why these viruses maintain different phenotypic variations in humans, thereby enabling a more accurate analysis of their relationship.

## Methods

### Viruses

The prototype strains of 12 HRV serotypes (HRV-3, 17, 27, 37, 52, 93, 11, 12, 24, 78, 64 and 94) were obtained from the American Type Culture Collection (LGC Promochem, Molsheim, France) and the RNA was eitherextracted directly from ATCC stocks (HRV-17, HRV-52, HRV-64 and HRV-94) orthe stocks were first amplified by one (HRV-11, HRV-12, HRV-24 and HRV-78), two (HRV-27) or three (HRV-3, HRV-37 and HRV-93) passages in HeLa Ohio cell lines (kindly provided by Prof FG Hayden, University of Virginia, Charlottesville, VA, USA). These serotypes were chosen tobe well scattered on the trees performed previously with HRV VP1 and VP4-VP2 subregions [16,22,23] and to complete sequence analysis of clinical isolates studied in the laboratory [2].

The full-length genome sequences of the 8 additional HRV serotypes (HRV-1B [GenBank:D00239], 2 [GenBank: X02316], 14 [GenBank:X01087], 16 [GenBank: L24917], 39 [GenBank:AY751783], 89 [GenBank:M16248], 85 and 9 whose sequences were directly downloaded from the *Picornaviridae *sequence database [29]), as well as the sequences of the 14 HEV serotypes and the simian picornavirus (SV-2) outgroup [GenBank:AY064708] analyzed in this study, were obtained from GenBand at the NCBI. The 14 HEV sequences include the two members of the HEV-D subspecies: EV-68 [GenBank: EF107098] and EV-70 [GenBank:DQ201177], the three members of the poliovirus subspecies: PV-1 [GenBank:V01148], PV-2 [GenBank:X00595] and PV-3 [GenBank: X00925] as well as three representatives of the HEV-A, B and C subspecies randomly chosen: Coxsackie (CV)-A2 [GenBank:AY421760], CV-A6 [GenBank:AY421764]and CV-A14 for HEV-A [GenBank:AY421769]; Echovirus (E)-1 [GenBank:AF029859], E-6 [GenBank:AY302558] and CV-B2 [GenBank:AF081485]for HEV-B; and CV-A1 [GenBank:AF499635], CV-A17 [GenBank:AF499639] and CV-A18 [GenBank:AF499640] for HEV-C. A list of all viruses with their corresponding GenBank accession numbers can be found in the additional file 8 in the supplementary material.

### Sequencing

Complete genome sequences were determined for each of the 12 above-mentioned strains. Reverse transcription (Superscipt II, Invitrogen, Basel, Switzerland) was performed with random hexamers on TRIzol- extracted (Invitrogen) RNA [60]. Overlapping fragments representing each complete viral genome were then amplified by PCR using degenerate primers designed to anneal highly conserved sequences among HRVs. Specific, non-degenerate primers were then designed to fill the gaps between the original PCR products. All primers used in this study are listed in the additional files (see additional file 7). The 5' and 3'ends were obtained with the 5'/3' RACE Kit (Roche Applied Science, Rotkreuz, Switzerland). PCR products were purified with the microcon columns (Millipore, Zug, Switzerland) before sequencing. Each PCR product was sequenced at least twice. Chromatograms produced with the ABI Prism 3130XL DNA Sequencer (Applied Biosystems, PE Europe BV, Basel, Switzerland) were directly imported for proofreading with the vector NTI Advance 10 program (Invitrogen).

### Multiple sequence alignment

Open reading frames (ORFs) were extracted from the whole-genome nucleotide sequences of each virus species using the getorf programme from the EMBOSS package [61], using a minimal ORF length of 6000 nt to ensure that small, spurious ORFs were not reported. The multiple alignment of encoded polyprotein was produced with the extracted ORFs using MUSCLE [62] with default parameters. The alignments for each of the protein products were extracted from the full multiple alignment. The whole-genome nucleotide level alignment was assembled using T-Coffee [63] from 3 separate alignments: 5'-UTR and 3'-UTR aligned with MUSCLE (default parameters), and the amino acid level multiple alignment of the ORFs projected to the nucleotide level using the TRANALIGN programme from the EMBOSS package with default parameters. The alignments are available from [64].

### Phylogenetic analysis

The maximum-likelihood phylogenetic analyses were performed using PhyML [65] with estimated proportion of variable sites, estimated Gamma distribution parameters and 16 substitution rate categories. Protein-level trees were made using the JTT [66] molecular evolution model, and nucleotide-level trees were made using the GTR model with empirical base frequency estimates. The consensus trees were reconstructed from bootstrap trees using PHYML or Tree-Puzzle [67] with the same parameters.

### Protein identity plots

All-against-all protein product identity scores were produced using the Belvu programme [68], and reformatted into symmetrical square arrays of sequence identity percentage values (one for each cleavage product) represented as greyscale bitmaps in Figures 2B and 2C.

### Bootscanning

A polyprotein multiple alignment was constructed (as described above) with 14 HEV sequences, 13 HRV-A sequences, 7 HRV-B sequences, and 1 SV-2 sequence. This alignment was subjected to bootscanning (as described in [69]) with a window size of 200 aa and a step of 50 aa. For each window, a maximum-likelihood tree with 100 bootstraps was computed as described above. HRV-A formed a single clade in all trees, HRV-B in all but one. HEV formed a single clade in many, but not all trees. The tree's topology was categorized as follows: i) the smallest clade that contained all rhinoviruses and at least one enterovirus was determined; ii) this clade was categorized as "HRV-B closest to HRV-A", "HRV-B closest to HEV", or "undecided" according to which clade was the sister clade of all HRV-B, iii) the bootstrap value of the clade determined in (i) was used as a measure of support of the topology. Finally, the log-likelihood of each tree was also recorded. For each window, this yielded: i) an indication of the most likely topology (with possibility of undecidedness); ii) a measure of support of this topology; and iii) a measure of confidence in the whole tree.

### GC content

We extracted sub-alignments for HEV, HRV-A, and HRV-B from the above-described nucleotide-level, whole-genome alignment of 14 HEV, 13 HRV-A, 7 HRV-B sequences. This allows direct comparison of the GC content at the orthologous positions using a sliding window of 600 nt along the alignment, computing GC percentage over all sequences within the window, and with a step of 10 nt. The resulting set of three measures of local GC percentage content, one each for HEV, HRV-A, and HRV-B were plotted.

### Identification of conserved RNA structural elements

The complete genome alignment of all 34 genomes spanning 7852 positions (5'UTR+ORF+3'UTR) was scanned for thermodynamically stable and structurally conserved RNA structures using RNAz [70] The structures were evaluated using a sliding window of 120 bp with 40 bp steps over the whole alignment, as well as separately for each of the three groups (HRV-A, HRV-B, HEV). To identify shorter secondary structure elements, the same procedure was performed using a window of 60 bp in steps of 20 bp. The consensus RNA structures of the selected alignment regions were folded using RNAalifold from the Vienna Package [71] with the least stringent option for consensus folding. These alignment regions were manually elongated and corrected in order to capture the whole RNA secondary structure. Furthermore, all alignment columns with more than 75% gaps were removed from the RNAalifold consensus folding procedure, since gaps are not excluded for the folding energies evaluation. The resulting structures as well as the alignments were color-coded according to the amount of consistent, compensatory and inconsistent base changes at a certain alignment and structure position using Vienna RNA Utilities [72].

## Abbreviations

HRV: Human Rhinovirus

HEV: Human Enterovirus

CV: Coxsackie Virus

E: Echovirus

PV: Poliovirus

EV-68: HEV-68

SV: Simian Picornavirus

*cre*: cis-acting replication element

UTR: untranslated region

UMP: Uridine monophosphate

IRES: internal ribosomal entry site

ORF: open reading frame

ML: maximum-likelihood

## Authors' contributions

CT designed the original project, conducted and supervised the experiments (primer design, PCR conditions, sequence assembly and proofreading) and drafted the manuscript. SVB conducted most of the experiments, SC conducted the *cre *sequencing and revised the manuscript. TJ conducted all the sequences, phylogenetic analyses and calculation of GC content and participated in the writing of the manuscript. DG analyzed the RNA secondary structure, identified the *cre *elements and participated in the writing of the manuscript. LP participated to the analysis and the writing of the manuscript. EZ supervised and designed all the bioinformatics work and corrected the manuscript. LK designed the original project, supervised the complete work and corrected the manuscript. All authors read and approved the final manuscript.

## Supplementary Material

Additional file 1 **Whole-protein maximum likelihood phylogenetic trees for the 11 individual picornavirus proteins**. Each individual protein tree was performed as the whole polyprotein phylogenetic tree (Figure 1). Click here for file

Additional file 2 **Similarity values among HRV-A, HRV-B and HEV for the 11 individual protein products**. The similarity values for protein comparison between HRV-A, HRV-B and HEV represented in Figure 2 are listed. Click here for file

Additional file 3 **5' UTR structure conservation**. A) 5'cloverleaf consensus structure for HRV-A, HRV-B and HEV identified by comparative sequence analysis. B) IRES consensus structure for HRV-A, HRV-B and HEV identified by comparative sequence analysis. See legend to Figure 3 for details. Click here for file

Additional file 4 **3'UTR structure conservation**. 3'UTR consensus structure for HRV-A, HRV-B and HEV identified by comparative sequence analysis. See legend to Figure 3 for details Click here for file

Additional file 5 **Conserved stem-loop structure in the ORF of HRV-A**. Conserved secondary structure located close to the 3'UTR of HRV-A and corresponding structures in HRV-B and HEV located in the same alignment region. See legend to Figure 3 for details Click here for file

Additional file 6 **Internal *cre *conservation among HRV-A and HRV-B serotypes**. A) Internal 2A *cre *conservation among HRV-A serotypes. B) Internal VP1 *cre *conservation among HRV-B serotypes Click here for file

Additional file 8 **Virus accession number**. List of all the accession numbers for the viruses used in the analyses. Click here for file

Additional file 7 **Primer list**. Degenerate and specific primers used to amplify and sequence the new rhinovirus genomes. Click here for file
