# Supplementary figures and images for: New complete genome sequences of human rhinoviruses shed light on their phylogeny and genomic features
Source: BMC Genomics. 2007 Jul 10;8:224. doi: 10.1186/1471-2164-8-224 (PMC1949831; doi:10.1186/1471-2164-8-224)

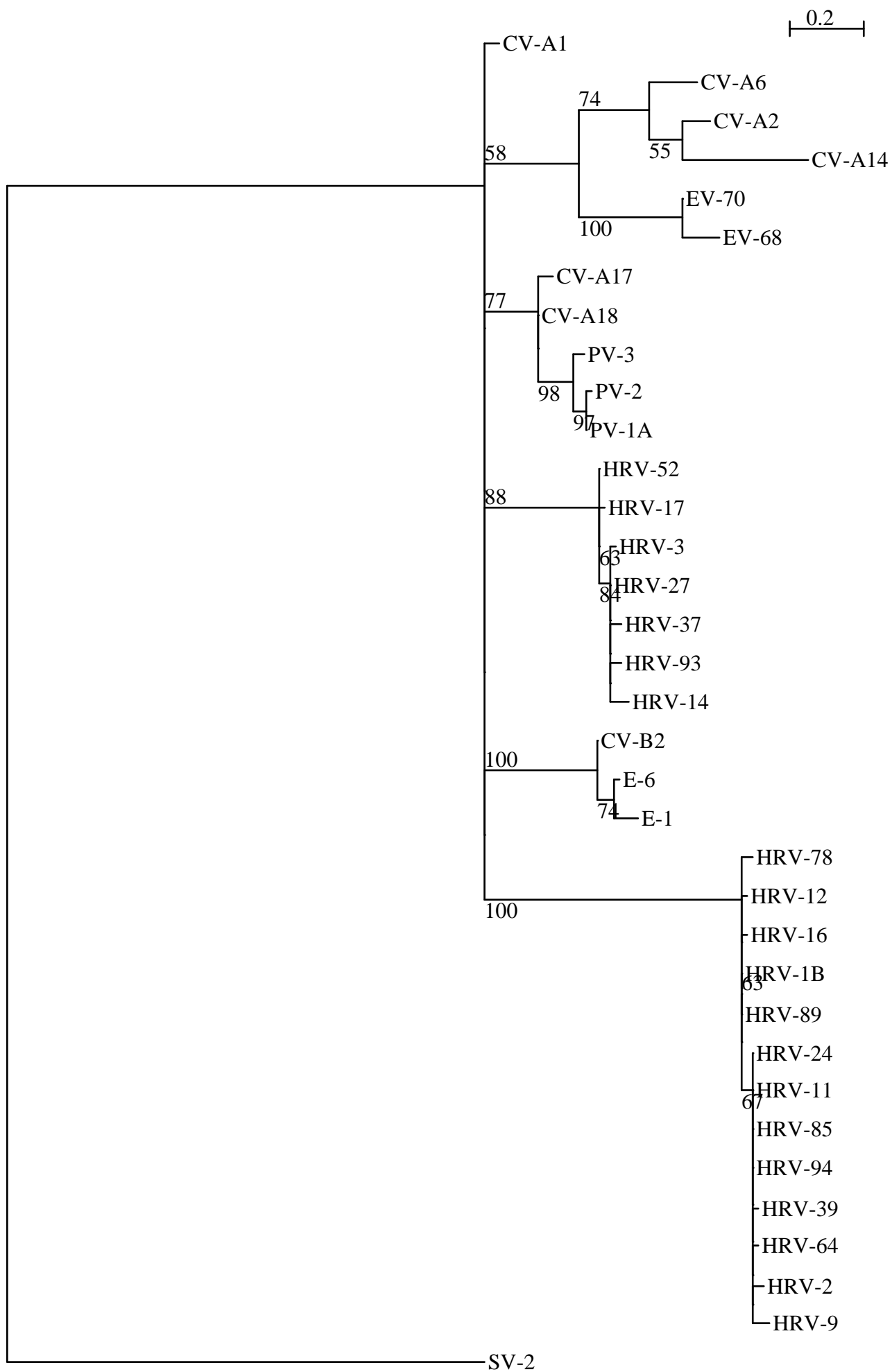

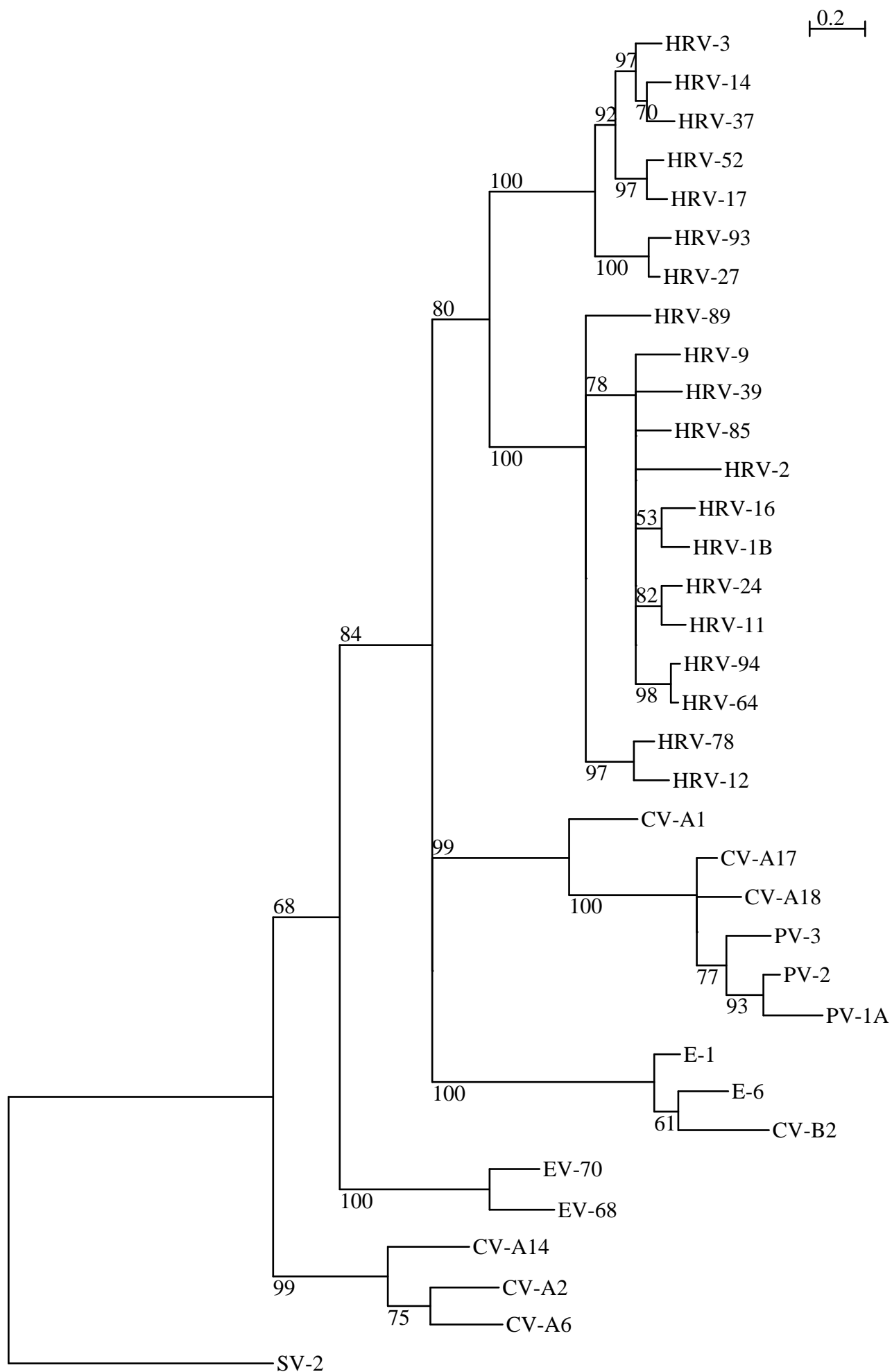

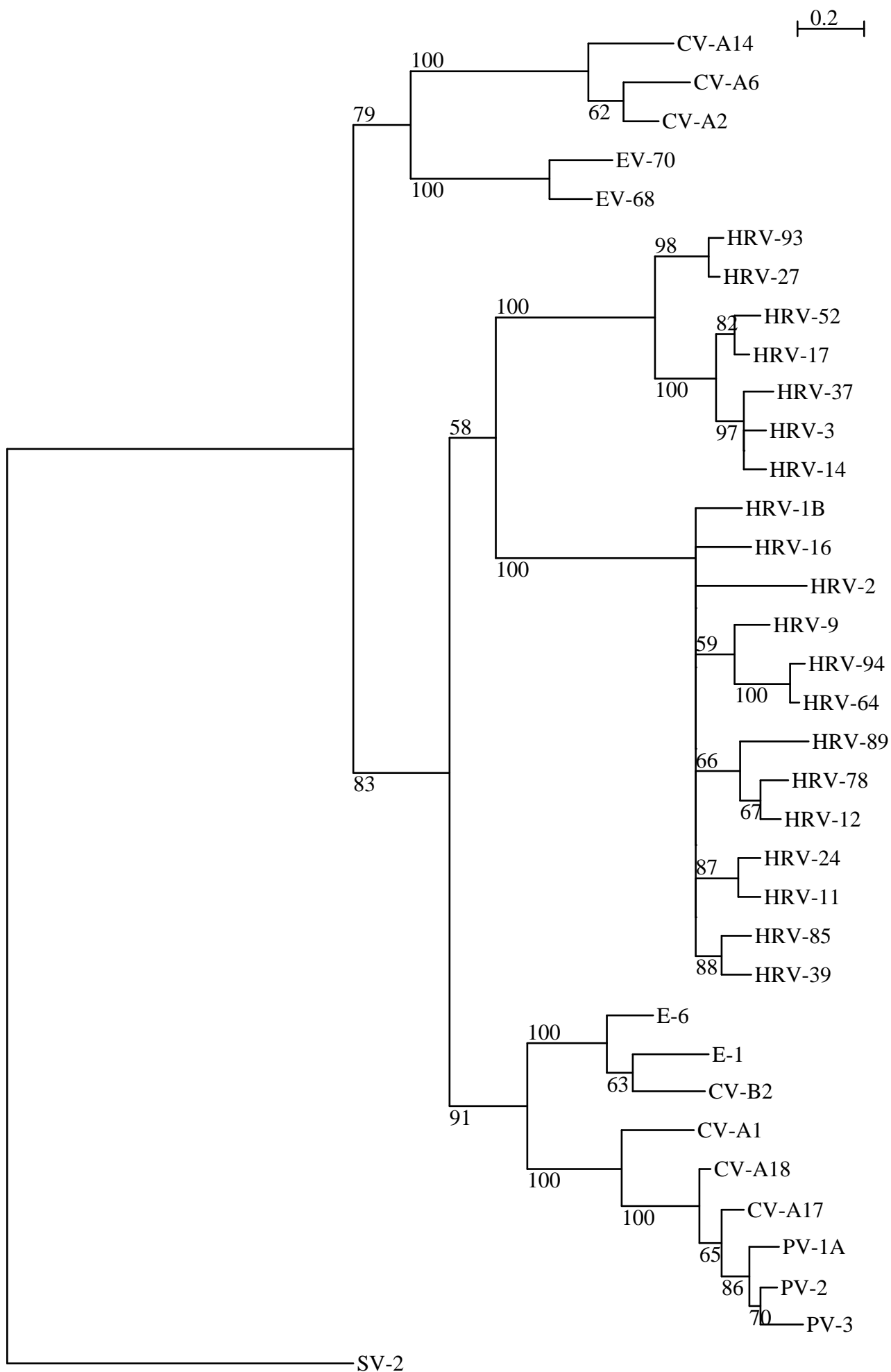

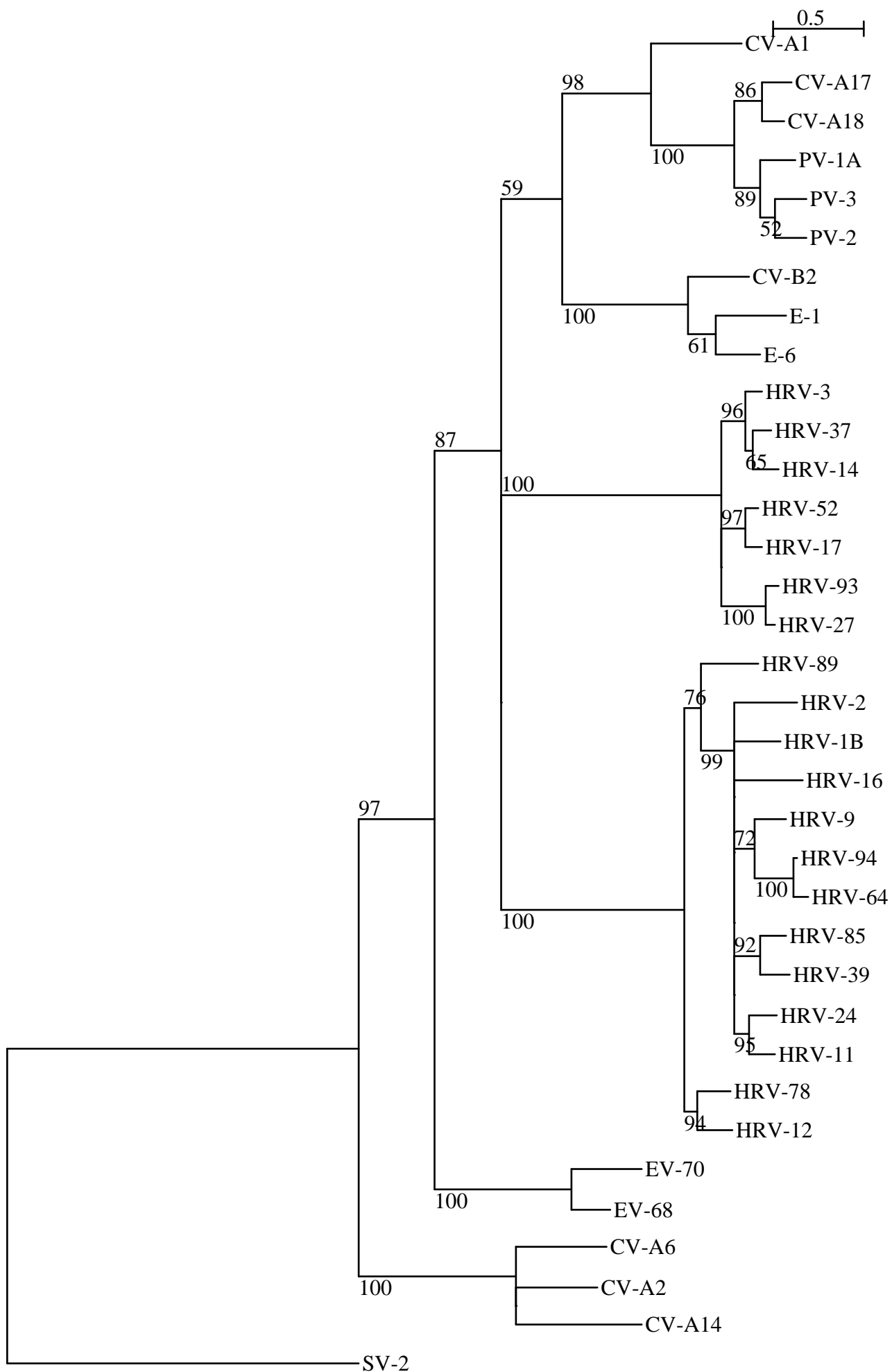

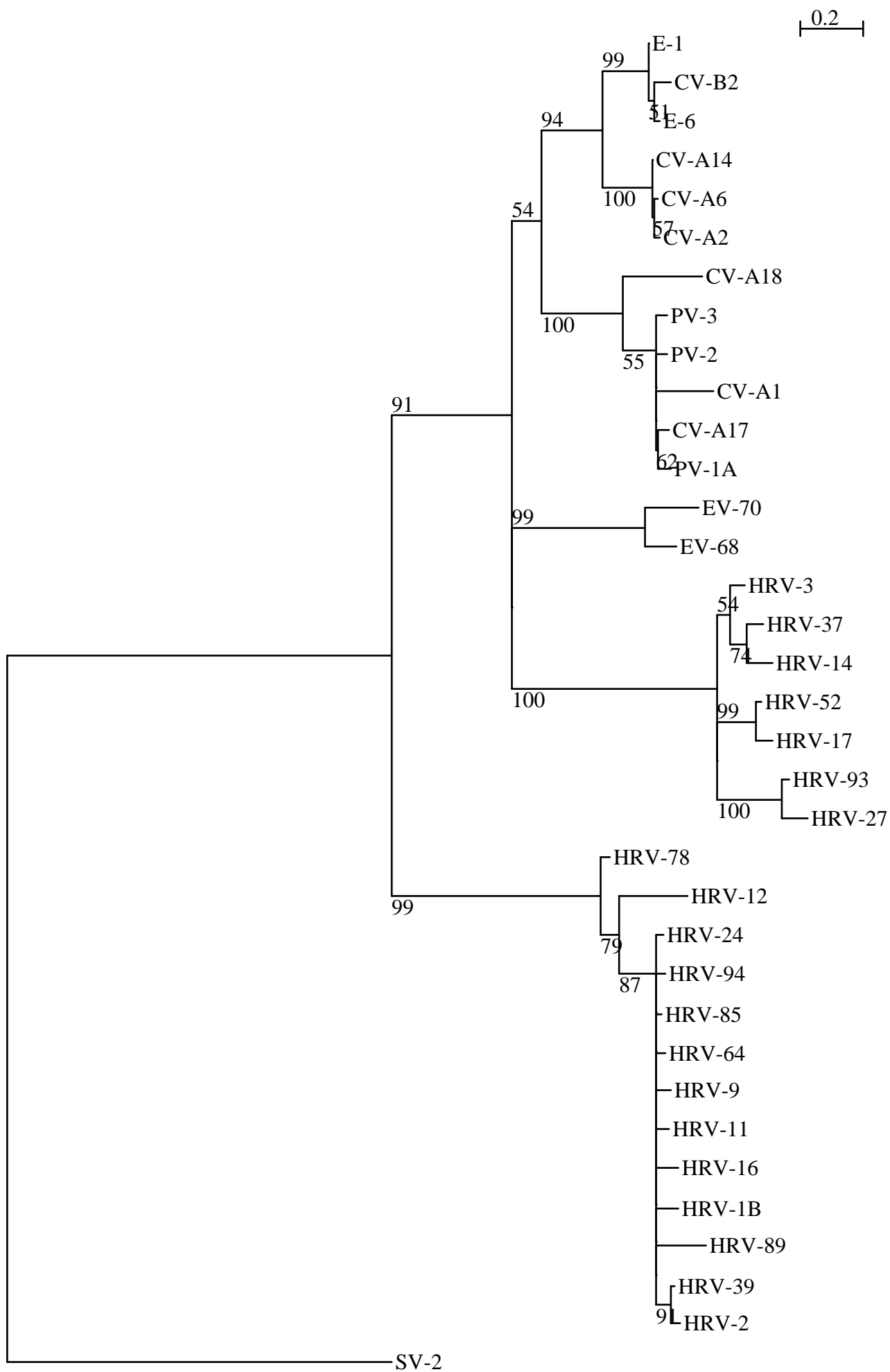

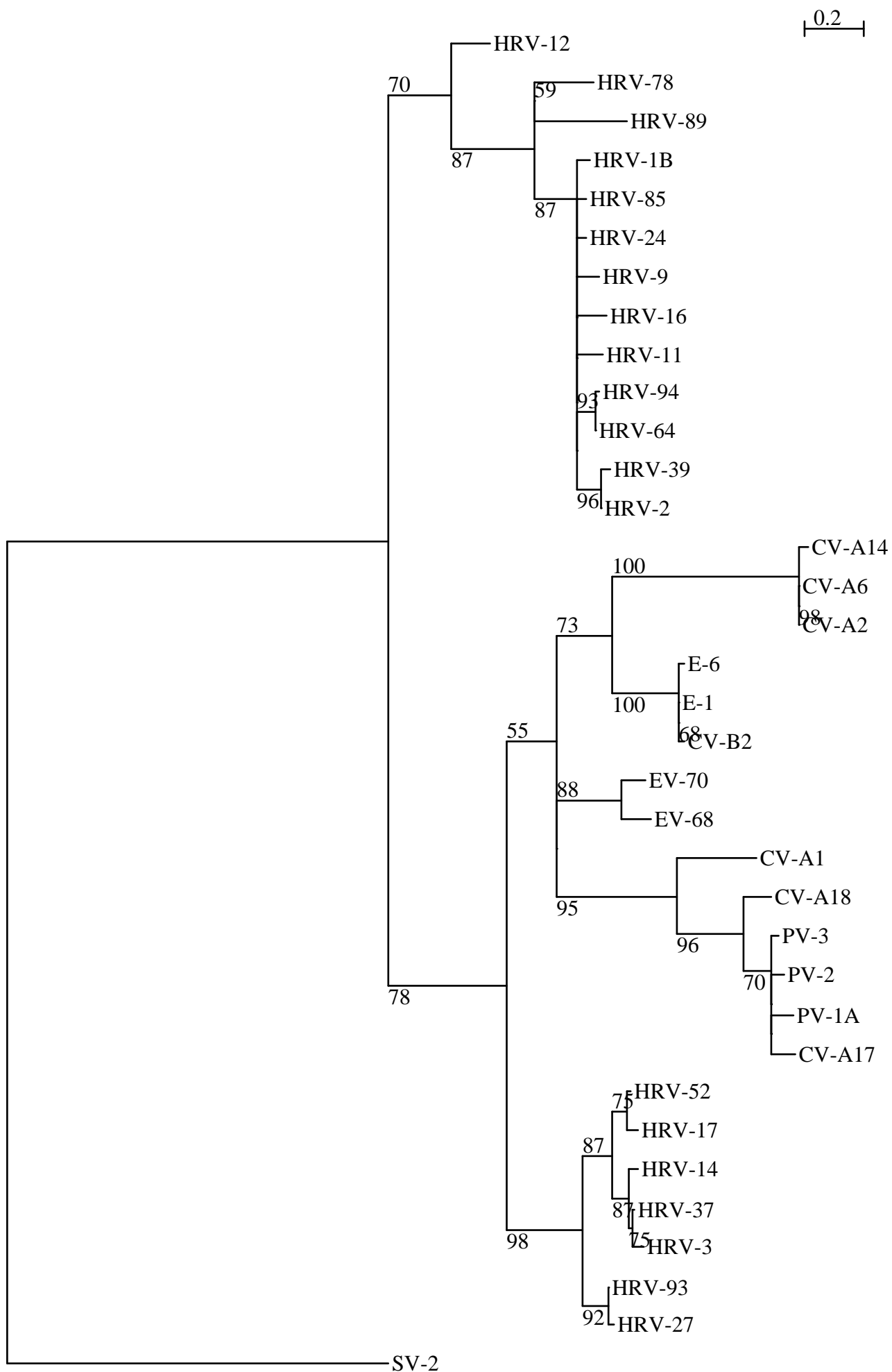

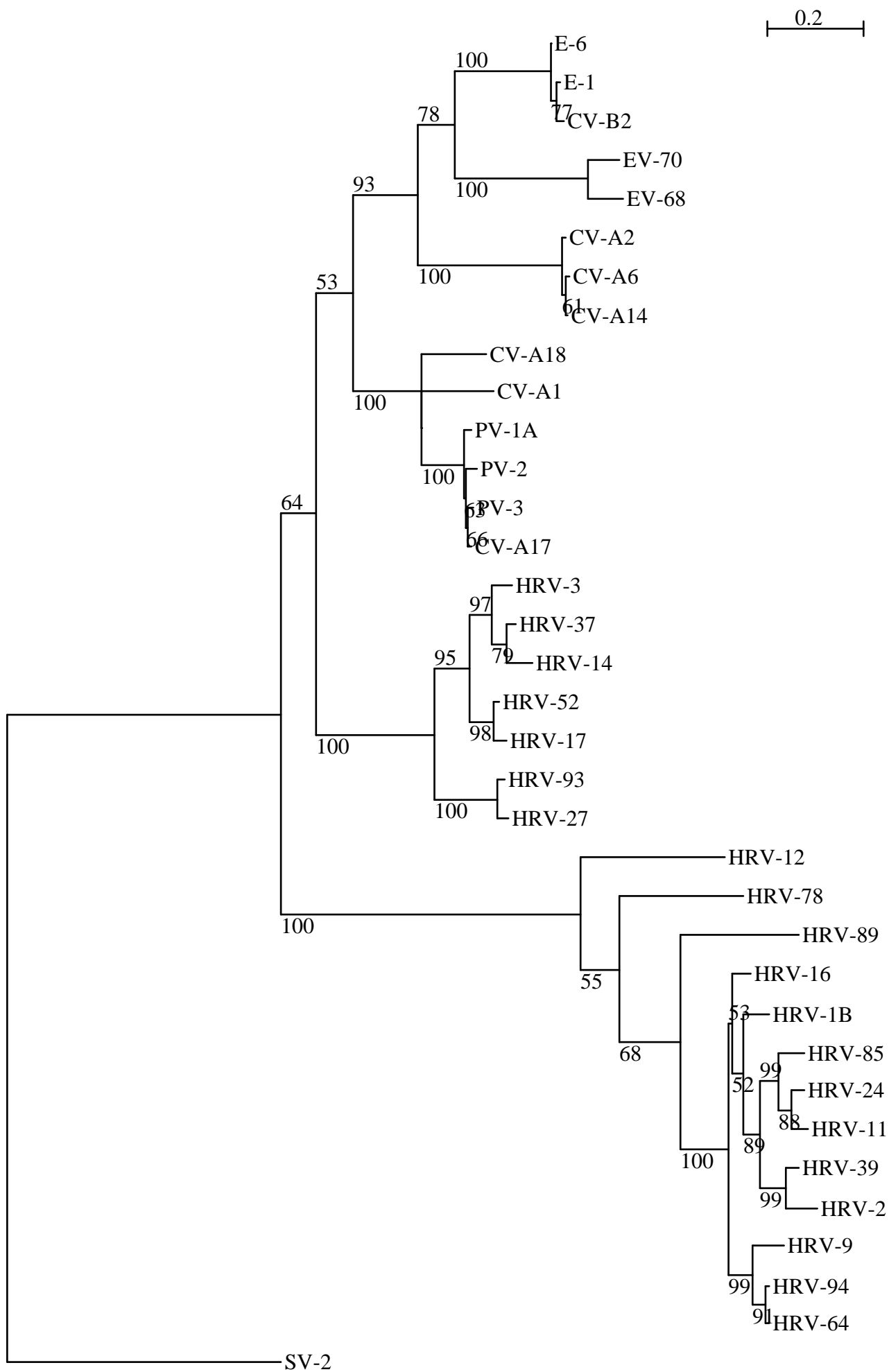

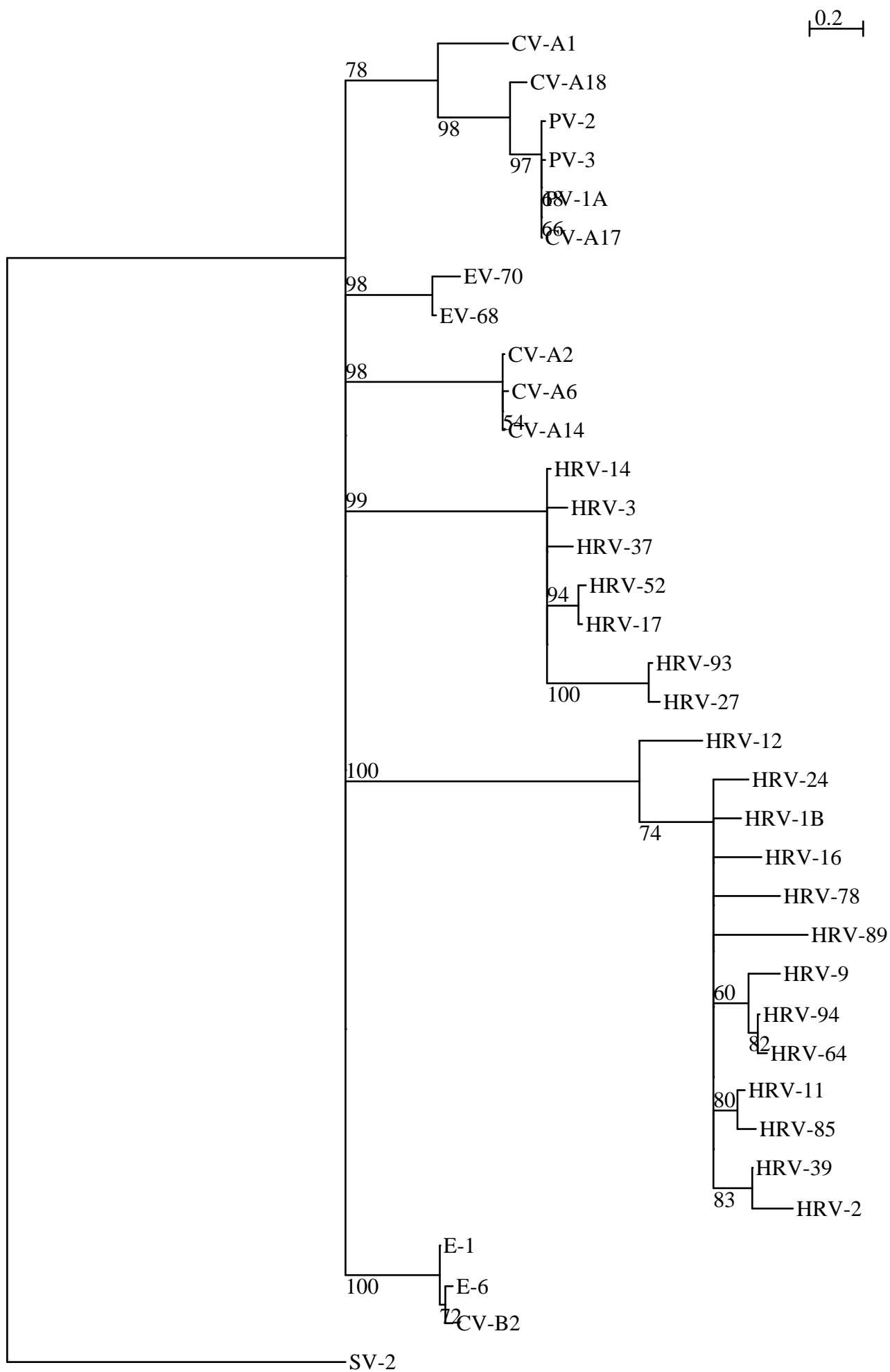

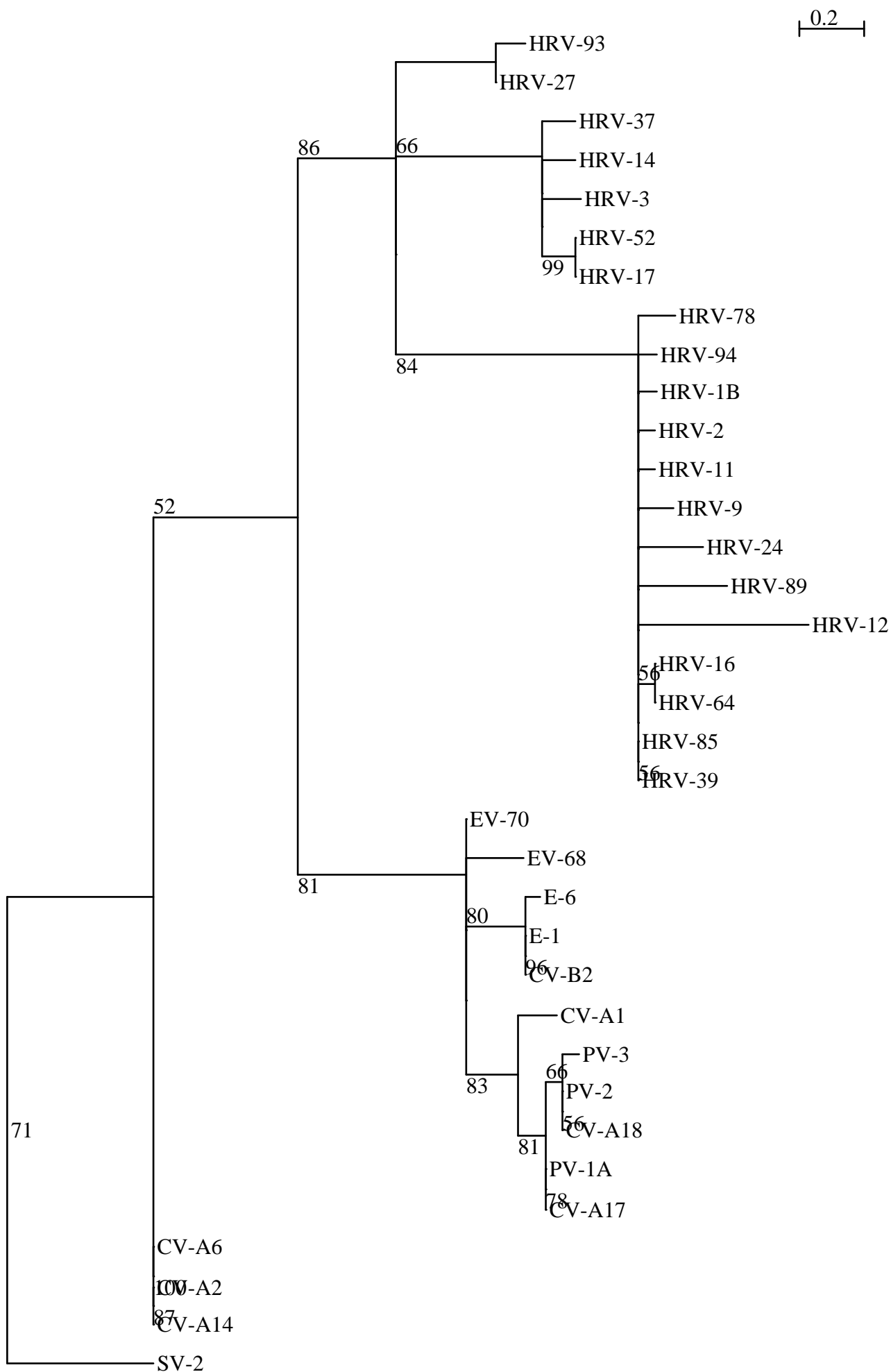

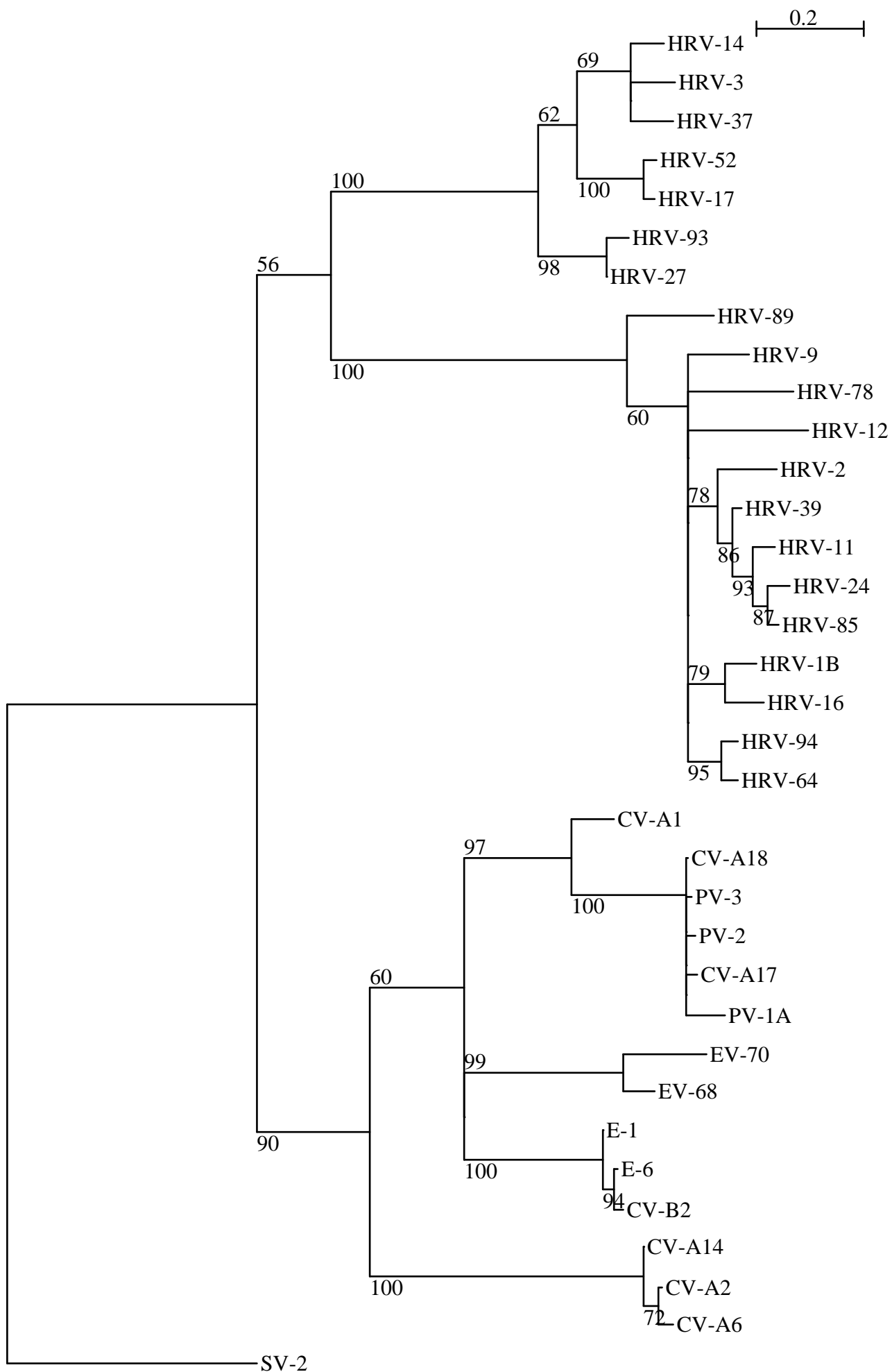

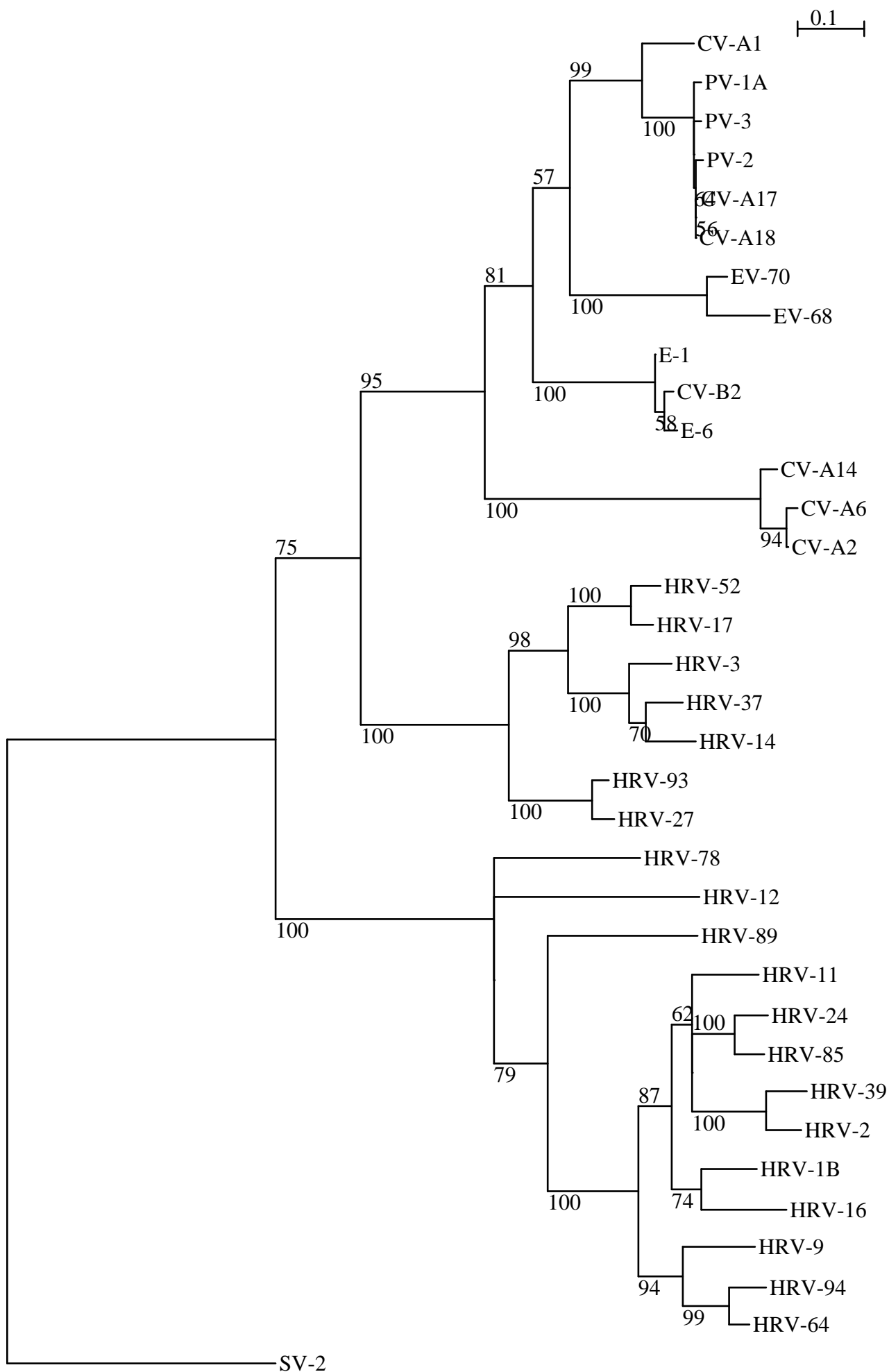

Supplement: Additional file 1 — Whole-protein maximum likelihood phylogenetic trees for the 11 individual picornavirus proteins. Each individual protein tree was performed as the whole polyprotein phylogenetic tree (Figure 1). [file 1471-2164-8-224-S1.pdf]

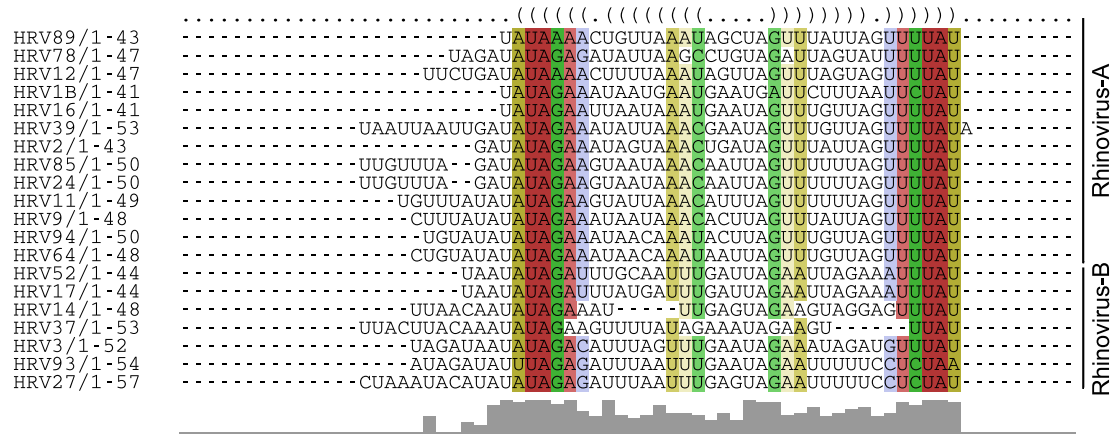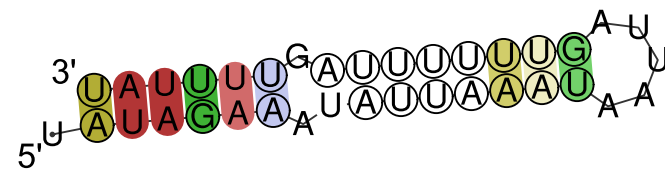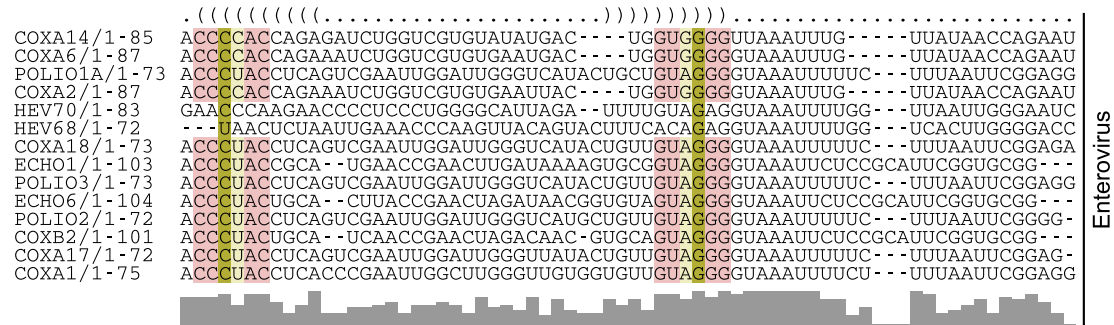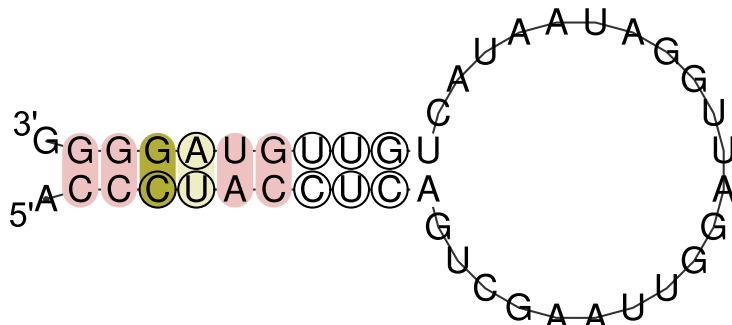

Supplement: Additional file 4 — 3'UTR structure conservation. 3'UTR consensus structure for HRV-A, HRV-B and HEV identified by comparative sequence analysis. See legend to Figure 3 for details [file 1471-2164-8-224-S4.pdf]

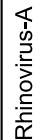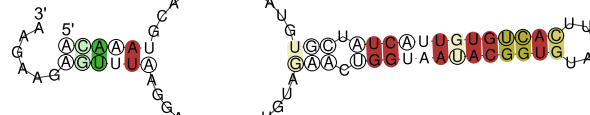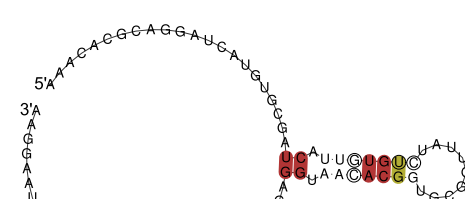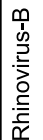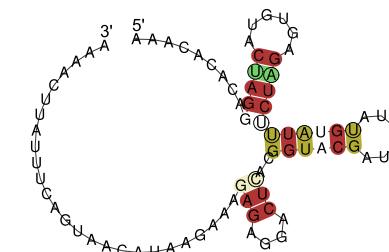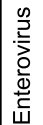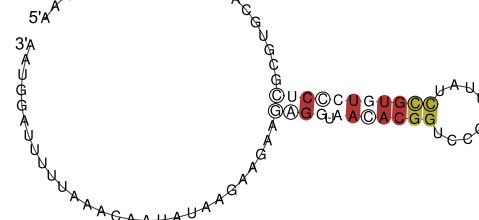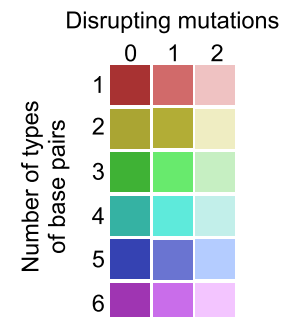

Supplement: Additional file 5 — Conserved stem-loop structure in the ORF of HRV-A. Conserved secondary structure located close to the 3'UTR of HRV-A and corresponding structures in HRV-B and HEV located in the same alignment region. See legend to Figure 3 for details [file 1471-2164-8-224-S5.pdf]

A)

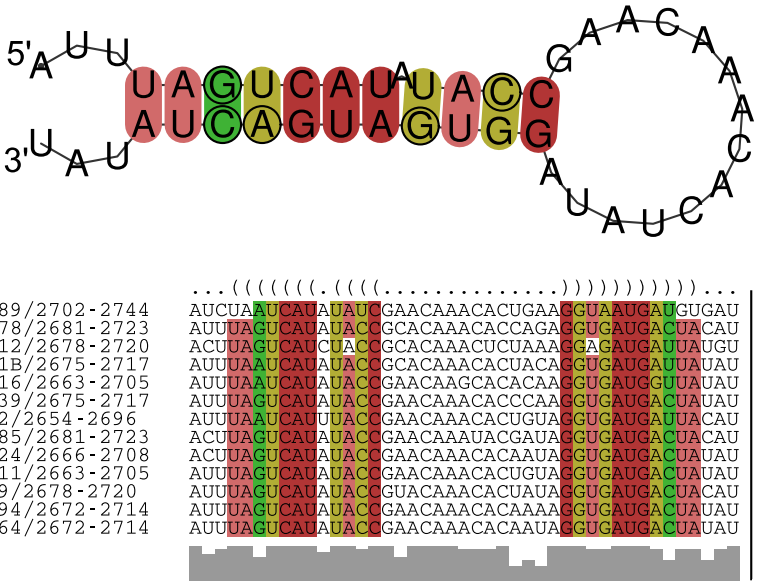

Disrupting mutations

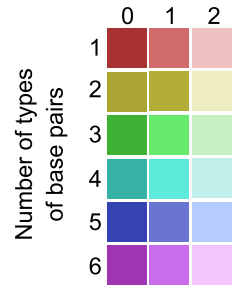

B)

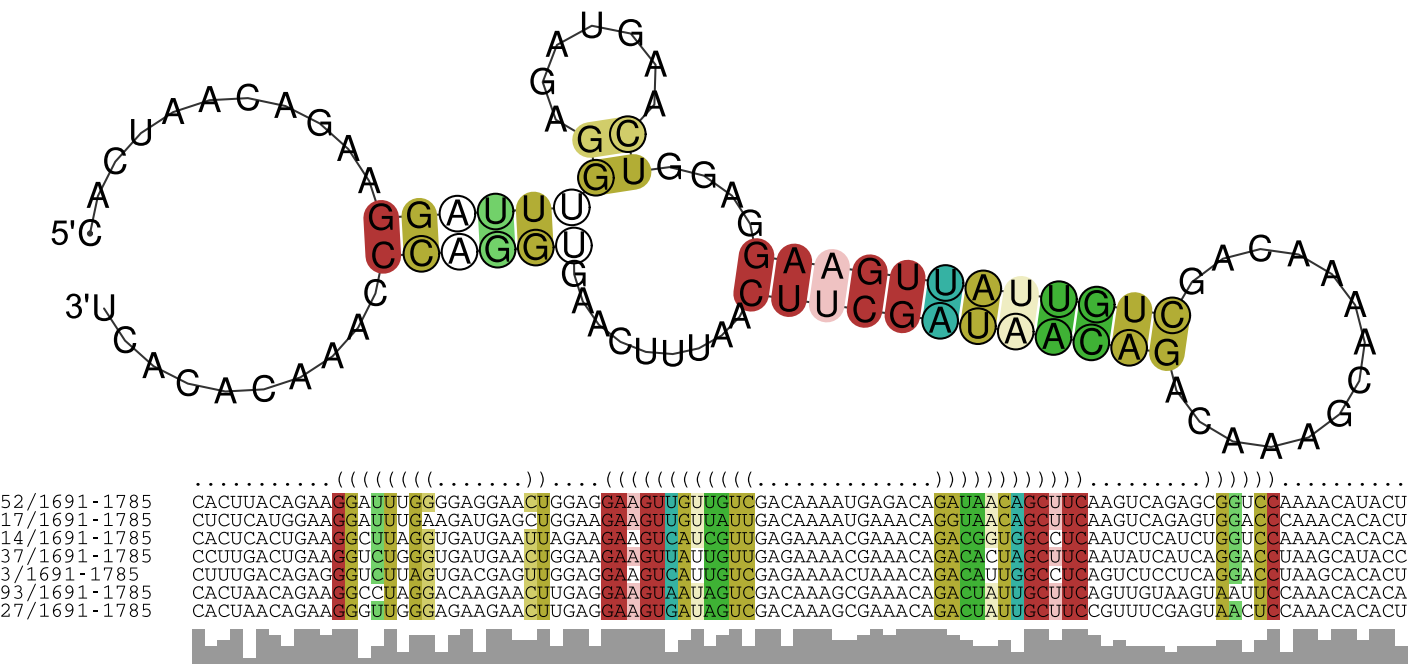

Supplement: Additional file 6 — Internal cre conservation among HRV-A and HRV-B serotypes. A) Internal 2A cre conservation among HRV-A serotypes. B) Internal VP1 cre conservation among HRV-B serotypes [file 1471-2164-8-224-S6.pdf]
