# Supplementary material for: New complete genome sequences of human rhinoviruses shed light on their phylogeny and genomic features
Source: BMC Genomics. 2007 Jul 10;8:224. doi: 10.1186/1471-2164-8-224 (PMC1949831; doi:10.1186/1471-2164-8-224)
Supplement: Additional file 3 — 5' UTR structure conservation. A) 5'cloverleaf consensus structure for HRV-A, HRV-B and HEV identified by comparative sequence analysis. B) IRES consensus structure for HRV-A, HRV-B and HEV identified by comparative sequence analysis. See legend to Figure 3 for details. [file 1471-2164-8-224-S3.pdf]

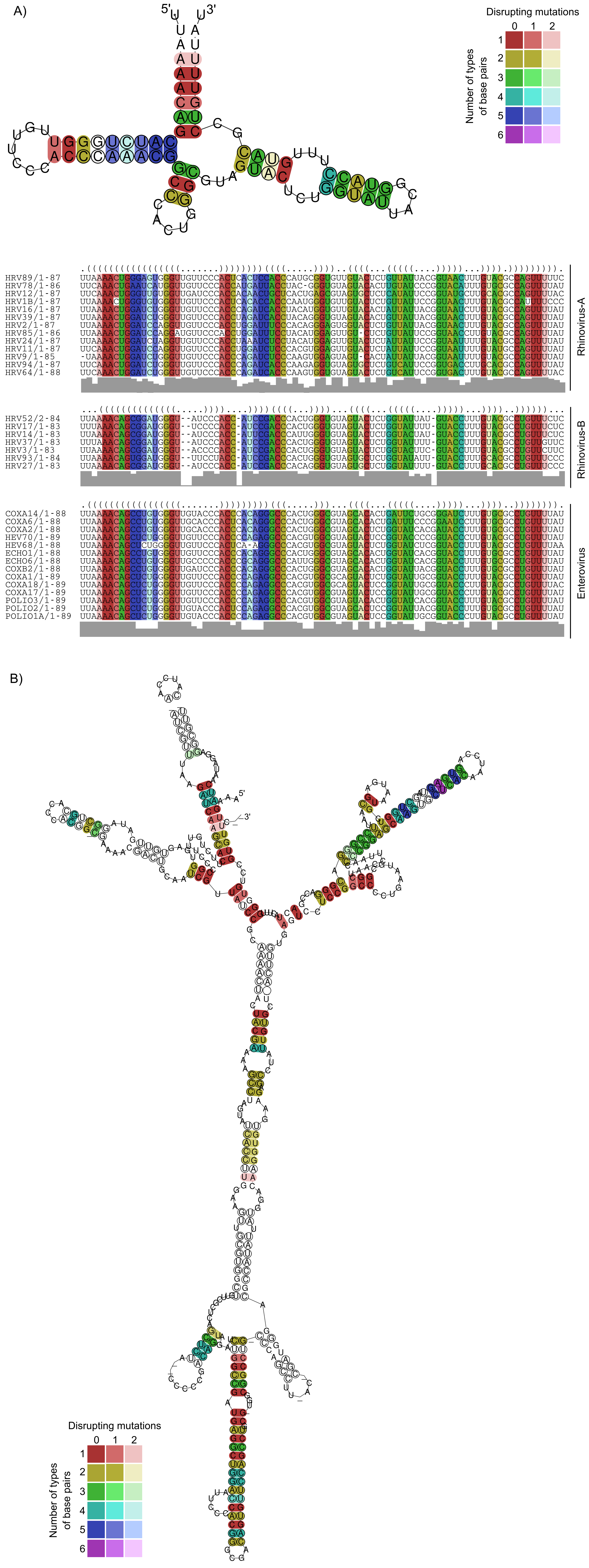

A)
5'
3'
Disrupting mutations
0 1 2
Number of types of base pairs
1
2
3
4
5
6
HRV89/1-87
HRV78/1-86
HRV12/1-87
HRV1B/1-87
HRV16/1-87
HRV39/1-87
HRV2/1-87
HRV85/1-86
HRV24/1-87
HRV11/1-87
HRV9/1-85
HRV94/1-87
HRV64/1-88
Rhinovirus-A
HRV52/2-84
HRV17/1-83
HRV14/1-83
HRV37/1-83
HRV3/1-83
HRV93/1-84
HRV27/1-83
Rhinovirus-B
COXA14/1-88
COXA6/1-88
COXA2/1-88
HEV70/1-89
HEV68/1-88
ECHO1/1-88
ECHO6/1-88
COXB2/1-88
COXA1/1-89
COXA18/1-89
COXA17/1-89
POLIO3/1-89
POLIO2/1-89
POLIO1A/1-89
Enterovirus
B)
5'
3'
Disrupting mutations
0 1 2
Number of types of base pairs
1
2
3
4
5
6
