# Supplementary material for: New complete genome sequences of human rhinoviruses shed light on their phylogeny and genomic features
Source: BMC Genomics. 2007 Jul 10;8:224. doi: 10.1186/1471-2164-8-224 (PMC1949831; doi:10.1186/1471-2164-8-224)
Supplement: Additional file 7 — Primer list. Degenerate and specific primers used to amplify and sequence the new rhinovirus genomes. [file 1471-2164-8-224-S7.doc]

**Table 1.** Primers used to amplify and/or sequence the genomes of the human rhinovirus prototype strains**.**

Position relative to HRV2

| **Sequence 5'-3'** | **Virus** | **Start** | **End** |
| --- | --- | --- | --- |
| CTACTTTGGGTGTCCG | HRV | 532 | 547 |
| CGGACACCCAAAGTAG | HRV | 532 | 547 |
| GGGACCAACTACTTTGGGTGTCCGTGT | HRV | 524 | 550 |
| GTACWCTRKTAYTMYGGTAMYYTTGTACGCC | HRV | 49 | 80 |
| GCACTTCTGTTTCCCC | HRV | 164 | 179 |
| ATTCAGGGGCCGGAGGA | HRV | 434 | 450 |
| CCGGGGAAACAGAAGTGCTTG | HRV | 161 | 181 |
| AGTGCATCTGGTAATTTCCA | HRV | 1055 | 1075 |
| TGGTGSTGGAARYTRCCWGATGC | HRV | 1049 | 1072 |
| GARCYWGGWGAYTGYGWGG | HRV | 3464 | 3484 |
| GGRTTYTGSWTNANRTCATCCAT | HRV | 4395 | 4417 |
| CCNCCWGAYCCNAARYAYTTTGATGG | HRV | 4322 | 4347 |
| TTIAGAAAYCTCCAIGGIGAICC | HRV | 3802 | 3825 |
| CARGGNCCWTAYTCNGG | HRV | 5066 | 5082 |
| CKNATRTCYYTRAAYTTYTCATTTCT | HRV | 5366 | 5391 |
| TCAWARTTWGWRTAATCAAANGCCAT | HRV | 6371 | 6396 |
| ACNAGTGCWGGNTWYCCNTAT | HRV | 6020 | 6040 |
| GGNTYCTTDGTCCATC | HRV | 6882 | 6898 |

**Table 2.** Strain-specific primers used to amplify and/or sequence the prototype strains of the indicated serotype.

Position relative to HRV2

| **Sequence 5'-3'** | **Virus*** | **Start** | **End** |
| --- | --- | --- | --- |
| AGCCTCATCTGCCAGGTCTA | HRV-A | 302 | 321 |
| CGTTAYCCGCAAGRYGCCTAC | HRV-A | 223 | 243 |
| TMAAACTGGATCTGGGTTG | HRV-A | 5'end | 50 |
| TGTKCGRTAWATGATTARATC | HRV-A | 3263 | 3283 |
| AATACTCATCTCATCTCTTGTTTG | HRV-64/94 | 2489 | 2512 |
| ATACAGATCACCAGAGGAGATT | HRV-78/12 | 857 | 878 |
| GACACTTACTCAAAAGCTGG | HRV-78/12 | 2141 | 2158 |
| AAGCAAATTCACTGATCCAG | HRV-78 | 760 | 779 |
| CCAGCATGTTATATACCCAGCT | HRV-78 | 2152 | 2173 |
| GAGACACACTTACTACAGTTGGACC | HRV-78/12 | 3141 | 3165 |
| CCTGTAAACAATGTGCAAGCTTCC | HRV-78/12 | 1760 | 1784 |
| GCAARYTGATGTTCTGGKATAGCA | HRV-78/64/94 | 1192 | 1215 |
| GCTTCAGYTTCATGTTTTGTGG | HRV-A | 1941 | 1962 |
| GATTTAGTCATMTACCGMACAAAC | HRV-A | 3263 | 3286 |
| GCAGCATTGCACATTTCTGT | HRV-A | 3896 | 3915 |
| AGAGGNCTTGAATGGATTGG | HRV-A | 3917 | 3936 |
| TTTCCWCCACAGTCWCCAG | HRV-A | 3468 | 3486 |
| CCAGCAGTAACTATTCCTATAACTCCATGT | HRV-24 | 3499 | 3528 |
| CCYTGTTCTTCAGCACARTGRAA | HRV-11/24 | 3566 | 3589 |
| GACCCTAAATACTTTGATGATGGATATGA | HRV-11/24 | 4328 | 4356 |
| GTTTGTCCGGTAGATGACTAAGTCCGA | HRV-78/12 | 3260 | 3286 |
| GATATATATTCCTTRCCACCAG | HRV-78 | 4307 | 4328 |
| TTTATCAGATTTATCTGCAGG | HRV-78/12 | 6743 | 6763 |
| CRCAYCCAACAGCWGARCC | HRV-A | 6299 | 6317 |
| ATGGATTGAATATTACACCTGCAG | HRV-12 | 6726 | 6749 |
| ATGGTCTTAAAATCACTCCAGCAG | HRV-78 | 6726 | 6750 |
| GGGTMCCATCAGGRTGTTCAGG | HRV-94/64 | 6531 | 6552 |
| TTTTTTTTTTTTTTTTATAAAACTAACAAAC | HRV-94/64 | 7336 | 3'end |
| TCAGCTGGAAAAACCCGTG | HRV-64 | 6185 | 6204 |
| GACAAGTATGGTGTTGATTTACCTATGGT | HRV-24 | 6116 | 6144 |
| GATAAATATGGAGTTGATTTGCCCATGGT | HRV-11 | 6116 | 6144 |
|  |  |  |  |
| CCTAGCCTCATCGACCAAACTA | HRV-B | 302 | 325 |
| GTGTCCTAGCCTGCGTGGC | HRV | 343 | 361 |
| CTAAACTTGTCTCTGTACCATC | HRV-27/93 | 2895 | 2917 |
| CCWRCACTRACWGCWAATGAAACTGG | HRV-B | 2405 | 2431 |
| TCATGCTCATTRACYAYYCTRAAAGCYAT | HRV-B | 2957 | 2986 |
| GATGGDAGKGTDGGAATDGTKGCCCC | HRV-B | 2429 | 2453 |
| TCAGRACAATTCCTDACYACRGATGA | HRV-B | 1628 | 1653 |
| GAATACCAGGGAATATCAGATTACAT | HRV-93 | 3578 | 3603 |
| ACAGTTGGAGGGTTGAGTGTCTG | HRV-93 | 4523 | 4546 |
| GAAGAATGTAAGCACATGTCTAA | HRV-93 | 4586 | 4609 |
| TGTRTACAYCTRAGTATVCCACCACA | HRV-B | 3476 | 3502 |
| GCTAAGGGWTTRGAATGGAT | HRV-B | 3914 | 3934 |
| GCTTTYCCACAWATYAGTGGACAACA | HRV-B | 4691 | 4716 |
| ATAATGAATTACCAYYTRATGACWCAWGA | HRV-B | 3200 | 3225 |
| AACAAGATATCAAAATTAATAGATTGGAT | HRV-B | 3947 | 3966 |
| ACAACAGACATYGGCAAATGT | HRV-3/37 | 5186 | 5206 |
| ATGGTCTGACTATCACACCACCAG | HRV-27 | 6726 | 6749 |
| GGGGAATTCACAGCCCTAGGAATCTATGACAG | HRV-93 | 5198 | 5229 |
| GGATCGCGNGTCCAKC | HRV-B | 6882 | 6898 |
| CCAGTGATGCCAATGCARGAYATT | HRV-17-52 | 6845 | 6869 |
| AAAGATAAATGGAGACTGGT | HRV-93 | 5300 | 5320 |
